# Evolution of Superficial Muscular Aponeurotic System Facelift Techniques: A Comprehensive Systematic Review of Complications and Outcomes

**DOI:** 10.1016/j.jpra.2023.06.003

**Published:** 2023-06-08

**Authors:** Hatan Mortada, Najla Alkilani, Ibrahim R. Halawani, Wasan Al Zaid, Rema Sultan Alkahtani, Hazem Saqr, Omar Fouda Neel

**Affiliations:** a Division of Plastic Surgery, Department of Surgery, King Saud University Medical City, King Saud University Medical City and Department of Plastic Surgery & Burn Unit, King Saud Medical City, Riyadh, Saudi Arabia; b King Saud University, College of Medicine, Riyadh, Saudi Arabia; c Faculty of Medicine, King Abdulaziz University, Jeddah, Saudi Arabia; d College of Medicine, Jouf University, Al Jouf, Saudi Arabia; e College of Medicine, King Saud University, Riyadh, Saudi Arabia; f School of Medicine, Newgiza University (NGU), Giza, Egypt; g Division of plastic surgery, Department of Surgery, King Saud University, Riyadh, Saudi Arabia and Division of Plastic Surgery, Department of Surgery, McGill University, Montreal, Canada

**Keywords:** Facelift, SMAS, Complications, Techniques, Aesthetic surgery, Facial nerve injury

## Abstract

**Background:**

Facelift procedures are a popular method of facial rejuvenation. The most common technique is superficial muscular aponeurotic system (SMAS) plication, with several variations. However, the optimal approach remains unclear. This review analyzed previous studies to compare SMAS facelift techniques, their outcomes, and complication rates.

**Methods:**

A systematic search was conducted using the MEDLINE, Cochrane, Embase, and Google Scholar electronic databases in September 2022. The search included studies published from January 2000 to September 2022 using keywords such as “facelift,” “complications,” and “outcomes.”

**Results:**

This review examined 27 selected studies that evaluated 6 SMAS facelift techniques. The studies involved 6086 patients in total, over 85% of who were satisfied with the outcome of their surgery. The complication rates varied depending on the technique used, with the SMAS flap and composite SMAS technique having the highest (5.75%) and lowest (0.05%) complication rates, respectively. The most common complications were temporary facial nerve injury (0.85%) and skin necrosis (0.41%). To date, only one case of permanent facial nerve injury has been reported.

**Conclusions:**

On the basis of our findings, SMAS facelift techniques achieve high patient satisfaction rates, with complication rates that vary by technique. The composite SMAS technique showed the lowest complication rates, whereas the SMAS flap showed the highest rate. However, some studies have not reported all complications, making it difficult to determine the best approach. Therefore, future studies are required to identify the most aesthetically pleasing technique with the lowest complication risk.

## Introduction

Facelift surgery is a procedure used to rejuvenate and reshape the face by manipulating its soft tissues rather than augmenting its bony prominences or underlying structures.^1^ This procedure was first modernized by French and German surgeons in the early 1900s; however, due to public perception, it was performed primarily in secret. During the Second World War in the 1940s, many soldiers were left with disfigurements on their heads and necks, prompting significant advances in plastic and reconstructive surgery. These improvements led to the development of newer facelift techniques pioneered by famous plastic surgeons such as Tord Skoog and Mitz and Peyronie, who developed the subfascial flap and superficial muscular aponeurotic system (SMAS) techniques, respectively.^1^, ^2^, ^3^

Today, various techniques are used in facelift surgery, including SMAS plication and lateral SMASectomy. A recently released study excerpt stated that SMAS plication is a popular technique for facial remodeling and rejuvenation because of its longevity, and the findings showed a decreased rate of complications.^4^ The lateral SMASectomy technique offers comparable results to other techniques, with another study concluding that it is a safe and adaptable method for facelifts.^5^

Previous systematic reviews analyzing the complications of facelift surgery have found that early relapse is an underreported complication. Most published studies on complications have included hematoma, seroma, necrosis, paresthesia, and poor scarring.^6^ A 2011 systematic review attempted to compare the efficacy and complication rates of different facelift techniques; however, the lack of quality data and evidence made it challenging to draw meaningful conclusions.^7^ A more recent systematic review identified significant differences in complication rates between techniques, although low incidence limited the clinical significance of the findings. However, this study was limited because not all relevant complications were listed in the data.^8^ Despite the popularity of the procedure, there is still much to learn about the different SMAS techniques and their potential complications. Given the significant number of people undergoing facelift surgery annually, a comprehensive understanding of the available techniques and their respective benefits and risks is crucial.

Most studies evaluating the complication rates of SMAS facelift techniques had a small pool of data, leading to inconclusive results that require further investigation. Therefore, this systematic review aimed to jointly analyze all previously published findings to compare different SMAS facelift techniques and their complication rates.

## Materials and Methods

### Literature search strategy

This study was registered in the International Prospective Register of Systematic Reviews (PROSPERO; protocol ID: CRD42022359268)^9^ and conducted according to the Preferred Reporting Items for Systematic Reviews and Meta-Analyses (PRISMA) guidelines and Cochrane Review methods.^10^ To conduct our systematic review, we performed a comprehensive literature search using electronic databases, including PubMed, MEDLINE, Embase, the Cochrane Central Register of Controlled Trials (CENTRAL), and Google Scholar. We limited our literature search from January 2000 to September 2022 to ensure that the techniques and complications discussed in the articles are relevant to current practices. By narrowing our search to this timeframe, we provided an up-to-date analysis of the current state of SMAS facelift techniques and their associated complications. The following search terms were used: “facelift surgery,” “SMAS facelift,” “rhytidectomy,” “rhytidoplasty,” “SMASectomy,” “complication rates,” “relapse,” “efficacy,” and “technique comparison.”

### Study selection criteria

We established the following inclusion criteria: (1) the publication was in English; (2) the study involved human participants who had undergone SMAS facelift surgery; (3) it reported on the incidence of complications associated with the facelift techniques; (4) the study had ≥ 20 adult patients; (5) it was based an original article, such as a prospective or retrospective cohort study, cross-sectional study, or randomized controlled trial; and (6) it was published between January 2000 and September 2022. We excluded studies that did not compare different SMAS facelift techniques or report on the incidence of complications. Review articles, case reports or series, economic analyses, cadaver studies, editorials, and conference abstracts were also excluded. Four authors independently screened the titles and abstracts of the identified articles and reviewed the full texts of the potentially eligible studies. The initial requirement was that the research provided a distinct explanation of a facelift technique that could be categorized as one of the following types: (1) SMAS plication, (2) SMAS excision (SMASectomy) or imbrication, (3) SMAS flap, (4) high lateral SMAS flap, (5) deep-plane, or (6) composite. Articles were extracted from the databases and screened using Rayyan (https://www.rayyan.ai/).^11^ In the cases where the title and abstract did not provide sufficient information, the full text was also reviewed. Any disagreements were resolved by discussion or through consultation with a fifth author.

### Data extraction

Data were extracted independently by 4 authors using a standardized data extraction form in a Microsoft Excel spreadsheet. The following data were extracted from each selected study: author, country, study design, sample size, mean patient age, sex, body mass index, comorbidities, type of facelift, temporary/permanent facial nerve injuries, skin necrosis, major/minor hematoma, seroma, infection, facial nerve injuries, long-term outcomes, and patient satisfaction. Any discrepancies in the data extraction were resolved by discussion or through consultation with a fifth author.

### Quality assessment and level of evidence

Quality assessment of the selected studies was performed using the Methodological Index for Non-Randomized Studies (MINORS).^12^ The MINORS is a validated tool for assessing the quality of non-randomized study methodology, including cohort, case-control, and comparative observational studies. It consists of 12 items for non-comparative studies and 8 for comparative studies, each scored on a 0–2 scale, with maximum scores of 16 and 24 for non-comparative and comparative studies, respectively. Two authors independently assessed the studies using the MINORS tool, and disagreements were resolved by discussion or through consultation with a third author. Studies were considered to be of high or low quality if they scored above or below the median score for their study type, respectively. In addition, we utilized the American Society of Plastic Surgeons’ (ASPS) levels of evidence and grading of recommendations to assess the quality of evidence for each study included in the review.^13^ The ASPS level of evidence categorizes studies based on the study design and quality of evidence, with Level I being the highest level of evidence and Level V being the lowest. The grading system assigns a strength of recommendation to each study based on the level of evidence, with Grade A being the highest level of recommendation and Grade C being the lowest. Two authors independently assessed the levels of evidence and grading of recommendations for each study. Any discrepancies were resolved by discussion or through consultation with a third author.

### Data analysis

We analyzed the data using a narrative synthesis approach, which involved descriptively summarizing the findings of the included studies. The results of each study were compared based on the type of SMAS facelift technique used, reported incidence of complications, and level of evidence provided by each study. Owing to significant heterogeneity in the study design, sample size, and reported outcomes, it was not possible to conduct a meta-analysis. Instead, we performed a qualitative synthesis of the data by summarizing the findings of each study and identifying trends or patterns in the reported results. Despite the lack of a meta-analysis, we believe that our narrative synthesis approach provides a comprehensive overview of the available evidence on the effectiveness and safety of different SMAS facelift techniques.

## Results

### Overview of the literature

**Figure 1:**
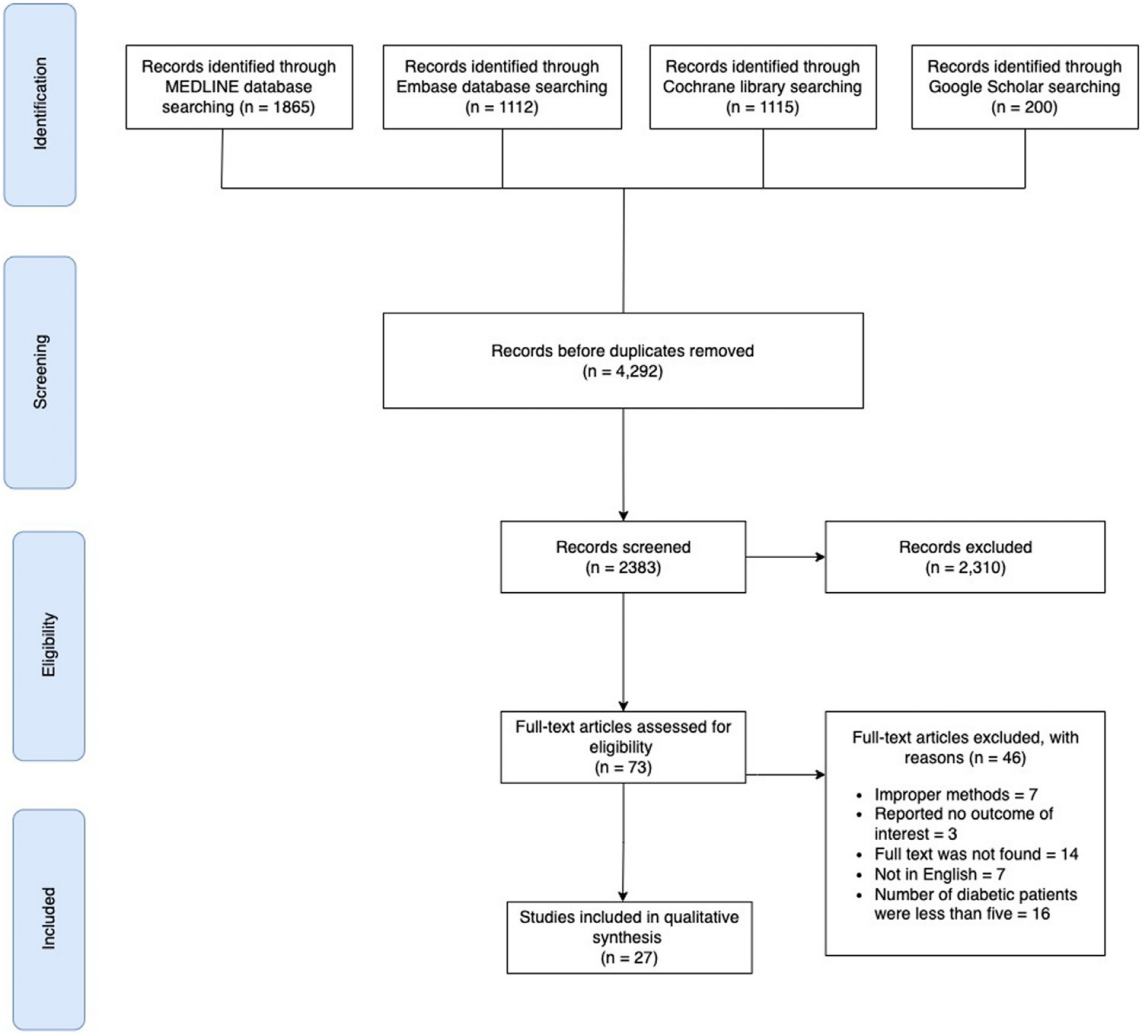
PRISMA flow diagram used to conduct this systematic review.

Initially, we identified 4292 articles, including 1865 from MEDLINE, 1112 from Embase, 200 from Google Scholar, and 1115 from the Cochrane library. The titles and abstracts of 2383 unique studies were screened after removing duplicate publications. Following the full-text screening of 73 remaining articles, 27 met our selection criteria.^14^, ^15^, ^16^, ^17^, ^18^, ^19^, ^20^, ^21^, ^22^, ^23^, ^24^, ^25^, ^26^, ^27^, ^28^, ^29^, ^30^, ^31^, ^32^, ^33^, ^34^, ^35^, ^36^, ^37^, ^38^, ^39^, ^40^ Reasons for exclusion included duplicate data in 5 studies, inappropriate methodology in 12 studies, 5 studies in a different language, a non-cosmetic facelift in 4 studies, a non-SMAS technique in 10 studies, and no outcomes of interest in a further 10 studies. All the selected studies were published between 2003 and 2021. Among the included articles, there were 23 retrospective and 4 prospective cohort studies. Twelve studies were conducted in the United States, 3 in the United Kingdom, 3 in China, and 2 each in Turkey and Chile. The other studies were from countries including Singapore, the Netherlands, Italy, Mexico, and Brazil. An overview of the PRISMA process used to conduct this systematic review is shown in Figure 1.

### Clinical characteristics of patients

**Figure 2:**
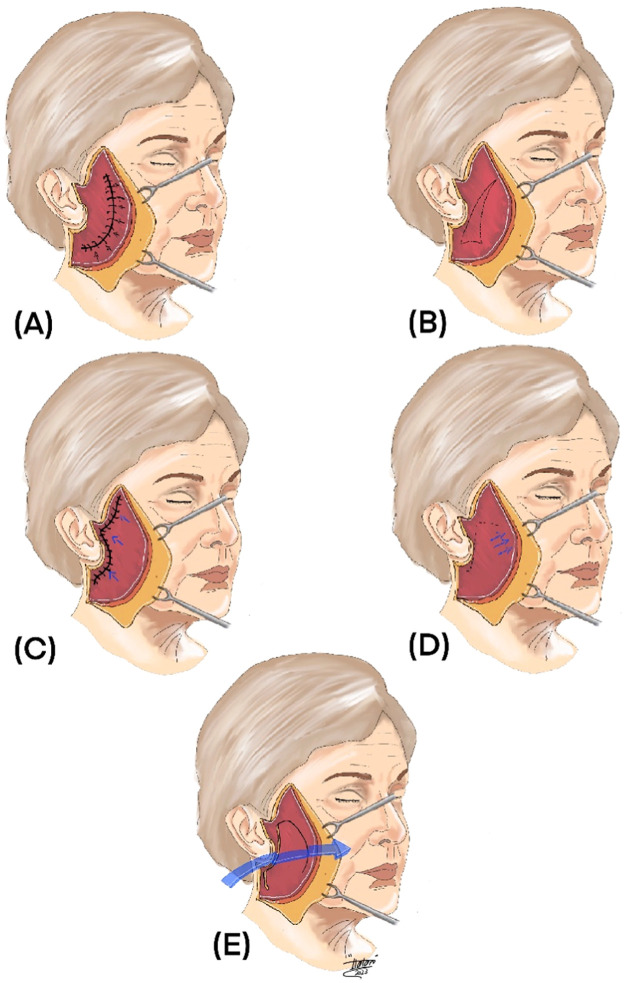
An illustration showing various SMAS facelift techniques. (A) SMAS plication: this technique involves folding and suturing the SMAS to create a lifting effect. (B) SMASectomy: in this approach, the SMAS layer is surgically excised or removed to decrease its volume, enabling repositioning and tightening of facial tissues. (C) SMAS flap: a flap of the SMAS is elevated and repositioned to achieve facial rejuvenation and lifting. (D) High lateral SMAS: the SMAS layer is dissected and lifted in an upward and outward direction, targeting sagging in the mid-face and jowls. (E) Deep plane facelift: this technique involves repositioning and lifting the deeper layers of facial tissues, including the SMAS, to achieve comprehensive facial rejuvenation.

Several different facelift techniques have been described in the literature (Figure 2); however, only studies that assessed SMAS facelift-related techniques were selected. Overall, 27 articles and a total of 6086 patients were included and classified into one of the 6 technique categories as follows: (1) SMAS plication, 7 studies and a total of 2309 patients; (2) SMASectomy, 4 studies and 604 patients; (3) SMAS flap, 11 studies and 2644 patients; (4) high lateral SMAS, 3 studies and 294 patients; (5) deep-plane vs. SMAS, one study and 107 patients; and (6) composite, one study and 128 patients.

Patient age ranged from 23 to 89 years with a mean of 55 years. Regarding sex distribution, 98 participants were male (4.7%) and 1984 (95%) were female. Most patients were medically free from comorbidities, or comorbidities were not mentioned. Hypothyroidism (n = 13), hypertension (n = 150), coagulopathy (n = 28), and diabetes (n = 12) were the most common comorbidities across the studies. Additionally, 82 patients who underwent facelifts were smokers. Adjunct procedures included upper blepharoplasty (n = 400), fat grafting (n = 362), lower blepharoplasty (n = 340), forehead lift (n = 174), neck lift (n = 131), laser resurfacing (n = 132), and canthoplasty (n = 1). In the current study, we only included research that reported SMAS facelift methods in patients who underwent the procedure for cosmetic reasons. For the evaluation of patient satisfaction, the most common assessment scale, used in 3 studies, was the FACE-Q. A variety of other assessment scales and methods were used to assess postoperative patient status. According to all the studies that assessed the level of satisfaction, > 85% of the participants were satisfied with the outcome.

### Comparison of complications and outcomes for SMAS facelift techniques

Complication rates of the different facelift techniques are summarized in Table 1. Temporary facial nerve injury was reported in 10 studies involving a total of 52 patients (0.85%). Only one study included patients with sensory (n = 11) or motor deficits (n = 9). Across all techniques, only one permanent facial nerve injury was reported. Skin necrosis was identified in 8 studies in a total of 25 patients (0.41%); the most common location was the tip of the posterior auricular region. Further, 49 patients who underwent SMAS plication (n = 2309) developed other complications (2.12%). Among them were 14 cases of hematoma, 5 cases of delayed wound healing, 6 of scarring, and 3 of seroma. In the 4 studies that used the SMASectomy technique in 604 patients, 57 developed the following complications (9.44%): 20 hematomas, 5 cases of scarring, 1 case of temporary neuropraxia, and 32 unspecified complications in one study. A total of 350 patients developed complications in the 11 studies that performed the SMAS flap technique (13%, n = 2644). In many of these studies, the number of complications was not specified; however, overall, 7 reported hematoma, 2 infection, 4 wound healing issues, one seroma, 2 alopecia, and one prolonged dysesthesia. Among the complications of the high lateral SMAS technique applied in 3 studies, complications were reported in 36 of the 294 patients (12). Two studies reported infections without the number of cases, whereas only one specified 4 infection cases, 5 cases of hematoma, 2 of seroma, 2 of scarring, one of ear pain, and 13 cases in which one or more of the following conditions occurred: dehiscence, temporary facial nerve weakness, necrosis, hematoma, additional procedure required, hypertrophic scar, palpable knot in the temporal region, and retro-auricular infection. Regarding long-term outcomes, most patients across the different techniques had satisfactory results without the recurrence of facial redundancy. In addition, the length of the follow-up varied between studies, with some patients requiring a follow-up for as long as 228 months. Table 1 shows the different follow-up durations.

**Table 1:** An overview of the included studies.

Authors	Journal	Study design	Country	Outcomes	Technique	Level of evidence
Becker^14^	*Archives of Facial Plastic Surgery*	R	USA	Surgical and anesthesia complications after rhytidectomy	SMAS plication vs. deep-plane	IV
Berry^15^	*Journal of Plastic, Reconstructive and Aesthetic Surgery*	P	UK	Cosmetic outcome/satisfaction, complications	SMAS plication	II
Bisaccia^16^	*Dermatologic Surgery*	R	USA	Achieving a minimally invasive face-lifting approach to the midface, cosmetic outcome, avoiding complications of traditional whole face-lifting procedures, downtime	SMAS plication	II
Calderon^17^	*Plastic and Reconstructive Surgery*	R	Chile	Cosmetic outcome satisfaction, complications	SMAS flap	IV
Chang^18^	*Plastic and Reconstructive Surgery*	R	USA	Outcomes of reoperative facelift using sub-SMAS techniques: no. of reoperative facelifts, interval between the previous and reoperative facelift, intraoperative findings of the SMAS-platysma anatomy, complications, rate of revision (a separate rate group was analyzed during the same period for the incidence of facial nerve injury only)	SMAS flap	IV
Hopping^19^	*The Annals of The Royal College of Surgeons of England*	R	USA	Complications, patient satisfaction	SMASectomy with or without imbrication	IV
Imber^20^	*Aesthetic Surgery Journal*	R	USA	Complications, patient satisfaction	SMAS plication	IV
Jones^21^	*Plastic and Reconstructive Surgery*	R	UK	Long-term outcome in a large series of consecutive facelifts performed with and without tumescence	SMAS flap	II
Jones^22^	*Journal of Plastic, Reconstructive and Aesthetic Surgery*	P	UK	Complications, aesthetic outcomes	SMAS plication	II
Leaf^23^	*Plastic and Reconstructive Surgery*	P	USA	Complications, patient satisfaction	SMAS flap	V
Lindsey^24^	*Annals of Plastic Surgery*	R	USA	Safety and effectiveness of SMAS flap dissection, extended SMAS flap mobilization and fixation	SMAS flap	II
Noone^25^	*Plastic and Reconstructive Surgery*	R	USA	Facial nerve injury, complications, prolonged convalescence and morbidities of extensive sub-SMAS or deep-plane dissections, nasolabial fold improvement, patient satisfaction	SMAS plication	IV
Ors^26^	*Journal of Craniofacial Surgery*	R	Turkey	Short- and long-term outcomes, patient satisfaction, possible complications of high SMAS facelift surgery under local anesthesia	High lateral SMAS	II
Prado^27^	*Plastic and Reconstructive Surgery*	R	Chile	Short- and long-term cosmetic outcomes of 2 minimal incision rhytidectomies and their advantages and disadvantages	SMASectomy with or without imbrication	II
Shauly^28^	*Aesthetic Surgery Journal: Open Forum*	R	USA	Complications, effectiveness of SMASectomy	SMASectomy with or without imbrication	III
Van der Lei B^29^	*Aesthetic Surgery Journal*	R	Netherlands and Canada	Patient satisfaction and complications associated with purse-string reinforced SMASectomy short-scar facelift	SMASectomy with or without imbrication	II
Wong^30^	*Plastic and Reconstructive Surgery*	R	Singapore and Australia	Satisfaction, aesthetic outcomes, complications, long-term outcomes	Composite	IV
Basile^31^	*Aesthetic Plastic Surgery*	R	Brazil	Effectiveness, safety, and complications of triple-anchoring sub-SMAS facelift	SMAS flap	IV
Bitik^32^	*Aesthetic Plastic Surgery*	R	Turkey	Effectiveness of sub-SMAS transposition of the buccal fat pad: average midface volume deficit score, average midfacial curvilinear surface measurements	SMAS flap	IV
Cárdenas-Camarena^33^	*Aesthetic Plastic Surgery*	R	Mexico	Effectiveness of cervicofacial rhytidectomy without notorious scars, patient satisfaction, complications, postsurgical morbidity, time to return to normal activity	SMAS plication	IV
Castello^34^	*Aesthetic Plastic Surgery*	R	Italy	Complications, patient satisfaction	SMAS flap	IV
Gong^35^	*Dermatologic Surgery*	R	China	Patient satisfaction, complications, effectiveness of lateral SMAS-stacking/SMASectomy; mean postoperative wrinkle, laxity, nasolabial fold depth, malar prominence, and tear trough deformity scores	High lateral SMAS	IV
Guyuron^36^	*Aesthetic Plastic Surgery*	P	USA	Patient satisfaction, efficacy of super-high SMAS facelift, complications	High lateral SMAS	IV
Rammos^37^	*Aesthetic Plastic Surgery*	R	USA	Complications, differences between patients who underwent subcutaneous facelift with or without SMAS plication and sub-SMAS facelift	SMAS flap	IV
Swanson^38^	*Plastic and Reconstructive Surgery: Global Open*	R	USA	Complications; scarring of the traditional facelift, which utilizes temporal incisions; facial volume	SMAS flap	II
Wang^39^	*Annals of Plastic Surgery*	R	China	Satisfaction and complication rates of combining SMAS plication, periauricular purse-string, and malar fat pad elevation technique for facial rejuvenation	SMAS plication	II
Zhou^40^	*Journal of Craniofacial Surgery*	R	China	Patient reported outcomes, complications, operation time, drainage volume, hospital days	SMAS flap	II

USA: United States of America; UK: United Kingdom; R: retrospective; P: prospective; SMAS: superficial muscular aponeurotic system

### Quality assessment and risk of bias

The quality of the included studies was assessed using the ASPS levels of evidence^13^ and grading of recommendations as well as the MINORS tool.^12^

ASPS levels of evidence and grading of recommendations: Of the 27 studies, 8 were categorized as Level III (evidence from case-control or cohort studies) and 19 as Level IV (evidence from case series or poor-quality cohort or case-control studies). None of the studies met the criteria for Level I or II.

MINORS: The MINORS tool was used to assess the quality of the non-randomized studies included in this review (Tables 2 and 3). The total scores ranged from 6 to 18, with a mean score of 12.3. The items with the lowest scores were the prospective calculation of the study size (score of 0 in all studies), unbiased assessment of the study endpoint (0 in all studies), and inclusion of a consecutive series of patients (1 or 2 in most studies). The items with the highest scores were the clearly stated aim of the study (2 in all studies), description of patient characteristics (2 in all studies), and clearly defined endpoints (2 in all studies).

**Table 2:** MINORS assessment of non-randomized non-comparative studies (n = 20).

Item	Berry^15^	Bisaccia^16^	Calderon^17^	Chang^18^	Hopping^19^	Imber^20^	Leaf^23^	Lindsey^24^	Noone^25^	Ors^26^	Castello^34^	Gong^35^	Guyuron^36^	Rammos^37^	Wang^39^	Shauly^28^	Van der^29^	Basile^31^	Bitik^32^	Cárdenas-Camarena^33^
A clearly stated aim	2	2	2	2	2	2	2	2	2	2	1	2	1	2	1	2	1	1	1	1
Inclusion of consecutive patients	2	0	2	0	0	0	2	2	1	2	1	2	2	2	2	2	1	2	1	1
Prospective collection of data	2	0	2	2	2	0	0	1	0	0	1	2	2	2	2	2	1	2	1	1
Endpoints appropriate to the aim of the study	2	2	2	2	2	2	2	2	2	2	0	2	2	2	1	2	1	2	1	1
Unbiased assessment of the study endpoint	2	0	0	0	0	0	0	0	0	0	0	2	0	0	0	0	0	0	0	0
Follow-up period appropriate to the aim of the study	0	2	2	2	2	0	2	2	2	1	1	0	0	0	2	2	1	2	2	2
Loss to follow-up less than 5%	0	0	0	0	0	0	0	0	0	0	0	2	0	0	2	2	2	0	0	0
Prospective calculation of the study size	0	0	0	0	0	0	0	0	0	0	0	2	0	1	0	0	0	0	0	0
*Total score*	10	6	10	8	8	4	8	9	7	7	4	16	7	9	10	12	7	9	6	6

**Table 3:** MINORS assessment of non-randomized comparative studies (n = 7).

Item	Becker^14^	Jones^21^	Jones^22^	Prado^27^	Swanson^38^	Zhou^40^	Wong^30^
A clearly stated aim	2	2	2	2	2	1	1
Inclusion of consecutive patients	2	2	2	2	2	1	2
Prospective collection of data	0	0	0	2	2	1	2
Endpoints appropriate to the aim of the study	2	2	2	2	2	1	1
Unbiased assessment of the study endpoint	0	0	0	1	0	0	0
Follow-up period appropriate to the aim of the study	2	2	2	2	1	2	2
Loss to follow-up less than 5%	0	0	0	2	0	2	0
Prospective calculation of the study size	0	0	0	0	1	1	0
An adequate control group	0	0	0	0	1	1	1
Contemporary groups	2	2	2	2	2	0	1
Baseline equivalence of groups	1	1	1	1	2	2	1
Adequate statistical analyses	0	2	2	0	2	2	0
*Total score*	11	13	11	16	17	14	11

Overall, the quality of the included studies was poor to fair, with a lack of high-level evidence and a high risk of bias in most studies. Therefore, caution should be exercised when interpreting the results of this review, and further high-quality studies are required to provide more reliable evidence.

## Discussion

This systematic review aimed to evaluate the incidence of complications associated with various SMAS facelift techniques. A total of 27 studies comprising 6086 patients were included. The most common complications were temporary facial nerve injury and skin necrosis. Other reported complications included hematoma, delayed wound healing, scarring, and seroma. One permanent facial nerve injury was reported across all included studies. The majority of patients who underwent different SMAS techniques had satisfactory long-term outcomes without recurrence of facial redundancy. Quality assessment and risk of bias results using the ASPS levels of evidence and grading of recommendations and the MINORS showed moderate quality of evidence for the included studies.

This study found that the overall complication rate of SMAS facelift techniques was relatively low (8.33%), with temporary facial nerve injury, hematoma, and skin necrosis being the most common. The incidence of temporary facial nerve injury was reported to be 0.85%, whereas 1.62% was reported for hematoma and 0.41% for skin necrosis. A single case of permanent facial nerve injury was identified. The SMAS flap technique had the highest overall complication rate at 13%, whereas SMASectomy had a complication rate of 9.44%. The high lateral SMAS and deep-plane techniques had complication rates of 12% and 11%, respectively. The lowest numbers of complications were observed for the SMAS plication (2.12%) and composite (2.34%) techniques. Although the studies varied in follow-up duration, most patients across all techniques had satisfactory long-term results without recurrence of facial redundancy. Evaluation of the long-term outcomes of SMAS facelift techniques was an important aspect of this study. Some patients required follow-up for up to 228 months, whereas others had shorter follow-up periods. This variation makes it challenging to draw definitive conclusions about the long-term effectiveness of these techniques.

Furthermore, some studies did not report any long-term outcomes, which limited this review. Future studies should have longer follow-up periods and report long-term outcomes to better evaluate the efficacy of these techniques over time.

Another important consideration is the potential effects of patient factors on long-term outcomes. For example, smoking and sun exposure reportedly negatively influence wound healing, which could impact the long-term success of facelift procedures.^41^ Future studies should control these factors to more accurately evaluate long-term outcomes.

The findings of this review have important implications for clinical practice. First, they demonstrate that SMAS facelift techniques achieved satisfactory outcomes with low complication rates. This finding supports the continued use of SMAS facelift procedures. Second, this review highlights the importance of careful patient selection and preoperative planning to minimize the risk of complications. Specifically, patients with significant medical comorbidities, smokers, and those with a history of facial surgery should be thoroughly evaluated and counseled about the potential risks and benefits of the procedure. In addition, surgeons should carefully consider the optimal SMAS technique based on the patient's individual characteristics and goals. Furthermore, this review emphasizes the importance of surgical techniques and skills in achieving successful outcomes and minimizing complications. Surgeons should have extensive training and experience in SMAS facelift techniques and continue to update their skills through ongoing education and training. Finally, this review highlights the need for standardized reporting of complications and outcomes across studies to facilitate comparison and improve the quality of evidence in this field. Overall, our findings provide valuable insights into the safety and efficacy of SMAS facelift techniques and may guide clinical decision-making and patient counseling.

### Limitations and future directions

Although this systematic review provides a greater understanding of the complications and outcomes associated with various SMAS facelift techniques, several limitations should be acknowledged. First, the heterogeneity in patient characteristics, techniques used, follow-up duration, and reported outcomes among the studies makes it difficult to draw definitive conclusions and generalize the findings across different populations.

Second, there is potential for publication bias among the studies because positive outcomes are more likely to be published than negative or inconclusive results. Additionally, the quality of the included studies varied, with some having a higher risk of bias than others.

Future research should focus on addressing these limitations and providing more robust evidence of the safety and efficacy of SMAS facelift techniques. Large-scale prospective studies with standardized protocols and longer follow-up durations are needed to generate more reliable data on the incidence of complications and long-term outcomes associated with these procedures. In addition, studies that directly compare different techniques would help identify the optimal approach for different patient populations. This study did not include a meta-analysis, which could have provided a quantitative synthesis of the results. Instead, we conducted a systematic review and narrative synthesis to summarize the available literature on SMAS facelift techniques and evaluate their quality. Although the meta-analysis provides more precise findings including estimates of effect sizes, it requires sufficient high-quality studies with similar outcomes and methodologies.

Future research should address these limitations by conducting well-designed studies with standardized outcome measures, longer follow-up periods, and detailed reporting of complications. Meta-analyses could be conducted once sufficient similar studies are available. Finally, studies that directly compare the effectiveness and safety of different SMAS techniques would provide valuable information for clinical decision-making.

## Conclusions

In summary, this systematic review evaluated the complications and long-term outcomes associated with different SMAS facelift techniques. The results suggest that the SMAS flap technique has the highest complication rate, whereas SMAS plication has the lowest. Temporary facial nerve injury, hematoma, and skin necrosis were the most commonly reported complications, whereas permanent facial nerve injury was reported in only one case. Overall, most patients treated using different techniques had satisfactory long-term outcomes without the recurrence of facial redundancy. However, this study had limitations, including the lack of a meta-analysis and the low to moderate level of evidence in most of the included studies. Future high-quality studies with larger sample sizes and longer follow-up periods should be conducted. Further research should compare the efficacy and safety of SMAS facelift techniques with those of non-SMAS facelift techniques to provide a more comprehensive understanding of the best overall approach for facial rejuvenation. Despite its limitations, this systematic review provides valuable insight into the complications and long-term outcomes of SMAS techniques. These findings could be useful for clinicians when selecting the most appropriate technique for a patient and informing him/her of the potential risks and benefits associated with these procedures.

## Ethical Approval

The need for ethical approval and informed consent was waived due to the nature of the study.

## CRediT authorship contribution statement

**Hatan Mortada:** Conceptualization, Formal analysis, Data curation, Investigation, Software, Writing – review & editing. **Najla Alkilani:** Conceptualization, Formal analysis, Data curation, Investigation, Software, Writing – review & editing. **Ibrahim R. Halawani:** Conceptualization, Formal analysis, Data curation, Investigation, Software, Writing – review & editing. **Wasan Al Zaid:** Conceptualization, Formal analysis, Writing – review & editing. **Rema Sultan Alkahtani:** Conceptualization, Formal analysis, Writing – review & editing. **Hazem Saqr:** Conceptualization, Formal analysis, Writing – review & editing. **Omar Fouda Neel:** Conceptualization, Formal analysis, Writing – review & editing.

## Declaration of Competing Interest

None.
